# Quantitative evaluation of radiodermatitis following whole-breast radiotherapy with various color space models: A feasibility study

**DOI:** 10.1371/journal.pone.0264925

**Published:** 2022-03-09

**Authors:** So-Yeon Park, Jin Ho Kim, Ji Hyun Chang, Jong Min Park, Chang Heon Choi, Jung-In Kim

**Affiliations:** 1 Department of Radiation Oncology, Veterans Health Service Medical Center, Seoul, Republic of Korea; 2 Institute of Radiation Medicine, Seoul National University Medical Research Center, Seoul, Republic of Korea; 3 Department of Radiation Oncology, Seoul National University Hospital, Seoul, Republic of Korea; 4 Biomedical Research Institute, Seoul National University Hospital, Seoul, Republic of Korea; 5 Department of Radiation Oncology, Seoul National University College of Medicine, Seoul, Republic of Korea; Universiti Teknologi Malaysia - Main Campus Skudai: Universiti Teknologi Malaysia, MALAYSIA

## Abstract

**Purpose:**

We analyzed skin images with various color space models to objectively assess radiodermatitis severity in patients receiving whole-breast radiotherapy.

**Methods:**

Twenty female patients diagnosed with breast cancer were enrolled prospectively. All patients received whole-breast radiotherapy without boost irradiation. Skin images for both irradiated and unirradiated breasts were recorded in red-green-blue (RGB) color space using a mobile skin analysis device. For longitudinal analysis, the images were acquired before radiotherapy (RT_before_), approximately 7 days after the first fraction of radiotherapy (RT_7days_), RT_14days_, and approximately 10 days after radiotherapy completion (RT_after_). Four color space models (RGB, hue-saturation-value (HSV), L*a*b*, and YCbCr models) were employed to calculate twelve color space parameters for each skin image. Skin dose measurements for irradiated breasts were performed using nanoDot optically stimulated luminescent dosimeters on the first fraction of radiotherapy. Subsequently, acute radiation dermatitis in each patient was assessed according to the Radiation Therapy Oncology Group scoring criteria at both RT_14days_ and RT_after_. Finally, several statistical analysis methods were applied to investigate the performance of the color space parameters to objectively assess the radiodermatitis.

**Results:**

Owing to radiation-induced skin damage, R value of RGB model as well as S and V values of the HSV model for irradiated breasts increased significantly, while those for unirradiated breasts showed smaller increases. These parameters showed the longitudinal changes in color space parameters within each group and between groups over time with statistical significance. Strong correlations of the parameters for irradiated breasts at RT_7days_ with skin doses and those at RT_after_ were observed with statistical significance.

**Conclusion:**

The R value of RGB model as well as the S and V values of HSV model showed relatively better performance in evaluating the acute radiation dermatitis. These color space parameters could therefore serve as useful tools to assess radiodermatitis severity in a dose-dependent manner.

## Introduction

Breast cancer is one of the most frequently occurring cancer types among women worldwide. According to recent statistics reported by the International Agency for Research on Cancer (IARC), breast cancer ranks as the second most common cancer type and is the fifth leading cause of all cancer-associated deaths in the world [1]. In 2012, approximately 1,670,000 incidences of new breast cancers were estimated, of which approximately 522,000 resulted in death [2–5]. With the remarkable technological advances being achieved in radiotherapy, this approach has gained a role in breast cancer treatment. The majority of breast-cancer patients have been treated via adjuvant radiotherapy after breast-conserving surgery or post-mastectomy, whereby the locoregional control rate and overall survival have improved [6, 7].

Although breast radiotherapy is known to significantly reduce breast cancer recurrence risks and mortality rates, it most commonly produces radiodermatitis as a side effect in patients [4]. Because the skin associated with or close to the primary tumor is exposed to a significant amount of radiation, these side effects cannot be avoided. Chronic radiation dermatitis results from epidermal basal layer damage due to radiation. This can be characterized by erythema and dry desquamation that can progress to moist desquamation and even skin ulceration and necrosis [8]. Depending on its severity, the damage may require treatment breaks and delays, which can adversely impact the cancer treatment [9, 10]. Timely prevention and treatment of radiodermatitis should be ensured for the patient to receive the full treatment effects and have a better quality of life. To this end, the severity of the radiodermatitis should be assessed and graded properly. In most previous studies, subjective visual assessments have been performed according to physician-assessed scoring criteria, such as the Radiation Therapy Oncology Group (RTOG) criteria, the National Cancer Institute Common Terminology Criteria for Adverse Events (CTCAE), and the World Health Organization (WHO) criteria, to determine the severity [8].

These physician-assessed scoring criteria are limited in that they are subjective and do not provide a quantitative measure, which result in considerable intra-investigator variations and inter-investigator differences [8, 11]. With the advances in radiotherapy plus immunotherapy and development of new therapeutic strategies for radiodermatitis, slight differences in skin damage are of interest, which renders objective assessments essential [12]. Consequently, various objective methods for chronic radiation dermatitis evaluation have been introduced. These methods utilize handheld devices such as reflectance spectrophotometers for the measurement of skin color in terms of the Commission Internationale de l’Eclairage (CIE) L*a*b* color space parameters [11–16]. In this three-dimensional color system, the L* value represents lightness (from 0: black to 100: white), and the a* and b* values represent the complementary red (>0)/green (<0) and yellow (>0)/blue (<0) color components, respectively. It was demonstrated that the L* values decreased (darker) and a* values increased (reddish) with skin damage by radiotherapy. However, the b* values did not change significantly [11–16]. Overall, better performance was achieved through this approach for evaluating acute skin damage, and remarkable correlations were observed between the physician-assessed scoring criteria and the objective parameters measured using the handheld devices [11–16].

Color space is a mathematical model used to represent color information as three or four different color parameters, followed by standardization of the color quantification. Various color spaces have been used for different applications including computer graphics, television broadcasting, image processing, and computer vision [17]. There exist many kinds of color spaces, such as the red-green-blue (RGB), hue-saturation-value (HSV), CIE L*a*b*, and luminance-based YCbCr models [18, 19]. Several studies have applied these varied color spaces for skin detection and segmentation, with notable performance being achieved [18–21]. Although various color spaces that can describe skin color and texture have been proposed for skin detection and segmentation, only the CIE L*a*b* color space has been used for the evaluation of radiodermatitis to the best of our knowledge. Therefore, in this study, we investigated the performance of various color space parameters to assess chronic radiation dermatitis in breast cancer patients by analyzing skin images acquired using a skin analysis device. We then examined the correlations of various color space parameters obtained from the skin images to measured skin doses and physician-assessed scorings of skin toxicity using the RTOG criteria. Finally, as part of the longitudinal investigation, we assessed the potential to predict the radiodermatitis severity early in the treatment course.

## Materials and methods

### Patient characteristics and treatment

We prospectively enrolled 20 female patients diagnosed with ductal carcinoma in situ between October 2018 and September 2020 at Seoul National University hospital. All the patients received whole-breast radiotherapy following breast-conserving surgery. The median age of the patients was 50 years (range: 40–68 years). The inclusion criteria were: Age > 20 years; no history of radiotherapy to treat breast cancer; presence of only unilateral breast cancer; no distant metastasis from breast cancer; no previous breast implants or reconstruction; and no recurrent breast cancer. None of the patients received previous or simultaneous chemotherapy. The characteristics and treatment factors of the patients are detailed in Table 1. This study protocol was approved by the Institutional Review Board of Seoul National University hospital (IRB No. D-1810-053-977) and was conducted in accordance with the Declaration of Helsinki. Informed consent was obtained from all patients prior to their participation in this study.

**Table 1:** Characteristics and treatment factors of patients (n = 20).

Clinical features	Patient number	(%)
Tumor site		
Left	8	40
Right	12	60
Radiotherapy technique		
FIF	12	60
IMRT	8	40
Energy		
6 MV	17	85
10 MV	1	5
6 + 10 MV	2	10
Chemotherapy		
Yes	0	0
No	20	100
Hormone therapy		
Yes	15	75
No	5	25

Abbreviations–FIF: field-in-field, IMRT: intensity modulated radiotherapy.

All patients were immobilized in the supine position on a breast board (CIVCO Radiotherapy, Coraville, IA, USA) with both arms placed overhead with the aid of arm support. The planning computed tomography (CT) images of the patients were acquired using the Brilliance CT Big Bore^TM^ (Philips, Amsterdam, Netherlands) with 3-mm slice thickness. The planning target volume (PTV) was of the ipsilateral whole breast. The PTV prescription dose was 40.5 Gy, delivered in 15 fractions by employing only 6-MV or a combination of 6-MV and 10-MV photons. None of the patients received boost irradiation to the tumor bed. Two-field tangential treatment plans for intensity modulated radiotherapy (IMRT) and field-in-field techniques were generated for left- and right-sided breast tumors, respectively. Moreover, all plans were normalized such that 95% of the prescription dose covered 100% of the PTV. In addition, the maximum PTV dose was to be < 110% of the prescription dose.

### Longitudinal evaluation of patients

Skin images of the irradiated breast were obtained at room temperature (25 ± 1°C) and under room light (200 ± 10 lux) using a mobile skin analysis device (API-100; Aram Huvis, Gyeonggi-do, Korea). This device utilized RGB white light in the normal mode with a spatial resolution of 1624 × 1212 pixels within a measurement area of 1 × 1 cm^2^. For acquisition of the skin images, all patients were instructed to assume the required treatment position: supine on a breast board with both arms held above their heads. Four measurement points for skin image acquisition were selected at the upper, lower, inner, and outer sides of the nipple in the irradiated breast, with a distance of 3 cm from the nipple. The surgical wounds were excluded from the measurement points. The first measurement procedures were performed before radiotherapy (RT_before_), and subsequent measurements were obtained approximately 7 and 14 days after the first fraction of radiotherapy (RT_7 days_ and RT_14 days_, respectively). The final measurements were obtained approximately 10 days after radiotherapy completion (RT_after_). The application of topical products such as emollient, cosmetics, and cleansers was not allowed up to 8 h prior to acquisition of the skin images. For each measurement, the same examiner measured the skin images to eliminate inter-examiner variation. Unirradiated breasts were also evaluated in the same manner as in the case of the control.

For skin dose measurements, we used the nanoDot optically stimulated luminescent dosimeter (OSLD) system (Landauer Inc., Glenwood, IL, USA), the performance of which was tested to increase confidence. On the first day of the breast radiotherapy, skin dose measurements were performed for each patient at the corresponding points of the irradiated breast for skin image acquisition. Three OSLDs in a group were located for each measurement point, and the dose values of those OSLDs were then averaged.

Subsequently, radiation dermatitis assessments for each patient were conducted according to the RTOG scoring criteria at both RT_14 days_ and RT_after_. The skin severities were classified from grade 0 to grade 4. To avoid potential inter-examiner variability, all patients were evaluated independently by the same experienced radiation oncologist who was blinded to the results obtained using the OSLDs and skin analysis device.

### Color space models

The skin images of both irradiated and unirradiated breasts acquired using the skin analysis device were recorded in RGB color space (24 bits per pixel) having three components corresponding to red (R), green (G), and blue (B). The three color components are generally quantized with 8 bits, and each color component has an integer value ranging from 0 to 255 representing the intensity of the color. The darkest color value is 0 and the brightness color value is 255 [18, 20]. We cropped each image into a circular region of interest (ROI) centered at the center of the image with a radius of 300 pixels. To extract the RGB color space parameters from the cropped skin images, the source code in MATLAB (version R2020a, Mathworks, Natick, MA, USA) was used. Each of the color space parameters was extracted from the cropped skin images in matrix type and then averaged as a representative value of the cropped skin images.

The HSV model is more intuitive than the RGB model regarding human perception of color, involving representation of the hue (H), saturation (S), and value of lightness (V). The HSV model color representation method is also based on human perception of color. H values range from 0 to 1, corresponding to the color’s position on a color wheel. As H increases from 0 to 1, the color transitions from red to orange, yellow, green, cyan, blue, magenta, and finally back to red. As S varies from 0 to 1, the corresponding colors (hues) vary from unsaturated (shades of gray) to fully saturated (no white component). As V increases from 0 to 1, the corresponding colors become brighter [18–20]. For parameter extraction, the RGB image was transformed into an HSV image using the ‘rgb2hsv’ command in MATLAB. Each parameter for the HSV model was calculated by averaging the corresponding converted HSV matrix.

The CIE L*a*b* color space model was specially designed to encompass all colors that the average human can perceive, based on color-opponent space [20, 21]. The L* values range from 0 to 100, where 0 specifies black and 100 specifies white. As L* increases, colors become brighter. Likewise, a* values that represent the amount of red or green tones in the images commonly range from –100 to 100; b* values representing the amount of yellow or blue tones in the images also range from –100 to 100. The cropped skin image was converted from the RGB mode to the L*a*b* mode using the MATLAB ‘rgb2lab’ function, via a process similar to that described above. The representative values for each L*a*b* parameter of the cropped skin images were obtained by averaging the corresponding converted L*a*b* matrix.

The YCbCr color space model is a digital color system for television transmission. In this model, the Y component represents luminance information, defined to have a nominal 8-bit range of 16 (black)–235 (white). On the other hand, the Cb and Cr components represent chrominance information as two color difference components, scaled to a nominal range of 16–240. Cb and Cr show the intensity of blue and red, respectively [18–21]. For parameter extraction, the RGB image was transformed into YCbCr image using the ‘rgb2ycbcr’ command in MATLAB, via a process similar to that described above. Each parameter for the YCbCr model was calculated by averaging the corresponding converted YCbCr matrix.

For each patient, a total of 384 color space parameters (12 color space parameters × 4 measurement points × 2 breasts × 4 longitudinal measurement points) were calculated in this study. For an improved understanding, S1 Appendix presents the detailed equations used for calculating the color space parameters for the different models.

### Statistical analysis

The significant longitudinal changes in the color space parameter values within each group (irradiated or irradiated breast) and the significant differences in those for the interactions with time and group were tested via a two-way repeated measures analysis of variance (RM-ANOVA). In the case of violation of sphericity assumptions (Mauchly’s test), the Greenhouse–Geisser correction was adopted to calculate the *F*-ratios. We considered *p*-values <0.05 as statistically significant. The Wilcoxon signed rank test with Bonferroni adjustment for multiple comparisons was used to assess pairwise comparisons of the color space parameter values between irradiated and unirradiated breasts for each of the longitudinal measurement points. In this case, *p*-values <0.0167 was considered statistically significant. To evaluate correlations between the color space parameter values and skin dose values measure using the nanoDot OSLDs, Spearman’s rank correlation coefficients and the corresponding *p*-values for all longitudinal measurement point were calculated. To test the performance of the color space parameters as a predictor of acute radiation dermatitis following radiotherapy completion, correlations of the color space parameter values for irradiated breasts at RT_after_ with those at RT_7 days_ and RT_14 days_ were determined using Spearman’s rank correlation. For these correlation tests, *p*-values <0.05 were considered as statistically significant. For all the statistical analyses, PASW Statistics 18.0 (SPSS, Chicago, IL, USA) was used.

## Results

### RTOG scoring and skin dose measurements

The severities of radiation dermatitis were assessed twice (RT_14 days_ and RT_after_) during the course of the experiment. With the RTOG scoring system, every patient with Asian ethnicity was scored under grade 1 at both RT_14 days_ and RT_after_.

The skin dose values measured using the nanoDot OSLDs for irradiated breasts are presented in Table 2. The mean dose delivered to the skin per patient was at least 214.9 cGy. In general, the majority of the skin doses for the inner side of the irradiated breast (14 cases) showed the lowest values, while those for both the upper and lower sides of the irradiated breast (17 cases) showed the highest values.

**Table 2:** Skin dose values measured using nanoDot optically stimulated luminescent dosimeters (OSLDs) for irradiated breast. Four measurement points were identified on the upper, lower, inner, and outer sides of the irradiated breast.

Patient ID	Upper (cGy)	Lower (cGy)	Inner (cGy)	Outer (cGy)	Mean (cGy)
1	260.2	251.7	221.8	266.3	250.0
2	271.4	245.5	244.0	233.8	248.7
3	256.0	247.4	202.8	284.0	247.6
4	272.2	229.5	238.1	199.3	234.8
5	262.2	231.0	240.5	213.1	236.7
6	265.2	237.7	212.6	253.7	242.3
7	262.9	232.1	208.4	223.1	231.6
8	228.5	224.7	186.7	219.8	214.9
9	264.6	227.9	209.5	236.6	234.6
10	247.4	217.3	209.7	204.9	219.8
11	249.3	275.0	200.9	217.6	235.7
12	240.4	243.0	203.5	248.9	234.0
13	264.0	274.6	261.8	250.9	262.8
14	246.1	273.7	214.4	202.1	234.1
15	255.6	265.1	199.7	223.9	236.1
16	232.9	244.2	204.4	212.4	223.5
17	223.1	273.1	189.1	203.5	222.2
18	267.9	263.4	198.4	226.4	239.0
19	259.0	252.7	193.7	201.9	226.8
20	234.0	246.0	207.2	213.7	225.2

### Evaluation of longitudinal changes in color space parameters

The mean and standard deviation values of the color space parameters for irradiated and unirradiated breasts are listed in Table 3. For irradiated breasts, the R value of RGB model, S and V values of HSV model, a* value of L*a*b* model, and Cr value of YCbCr model increased over time, while the other values of the models decreased over time. Although the b* value of the L*a*b* model and Cb value of the YCbCr model showed increasing and decreasing tendencies, respectively, they exhibited opposite trends until RT_7 days_. For unirradiated breasts, the corresponding color space parameter showed tendencies similar to those for irradiated breasts except for the a* value of the L*a*b* model. However, its changes in values over time were smaller than those for irradiated breasts.

**Table 3:** Mean and standard deviation values of color space parameters for irradiated and unirradiated breasts.

	Irradiated breast				Unirradiated breast			
	RT_before_	RT_7 days_	RT_14 days_	RT_after_	RT_before_	RT_7 days_	RT_14 days_	RT_after_
RGB(R)	188.822 ± 6.293	192.193 ± 6.447	196.722 ± 7.493	200.269 ± 10.087	186.020 ± 4.987	185.867 ± 4.514	186.511 ± 5.724	187.507 ± 6.838
RGB(G)	175.696 ± 3.585	173.131 ± 4.080	171.025 ± 3.752	168.874 ± 6.598	176.968 ± 2.062	176.333 ± 2.050	176.277 ± 2.301	176.800 ± 2.403
RGB(B)	139.622 ± 9.179	139.337 ± 7.365	137.071 ± 9.272	132.772 ± 10.386	142.547 ± 7.997	140.449 ± 8.384	138.181 ± 8.458	139.330 ± 9.536
HSV(H)	0.132 ± 0.030	0.115 ± 0.022	0.104 ± 0.014	0.096 ± 0.024	0.141 ± 0.024	0.139 ± 0.017	0.138 ± 0.016	0.136 ± 0.024
HSV(S)	0.264 ± 0.062	0.277 ± 0.054	0.304 ± 0.065	0.336 ± 0.077	0.239 ± 0.053	0.250 ± 0.053	0.263 ± 0.058	0.261 ± 0.066
HSV(V)	0.743 ± 0.021	0.755 ± 0.023	0.772 ± 0.028	0.786 ± 0.039	0.733 ± 0.016	0.733 ± 0.014	0.734 ± 0.020	0.739 ± 0.023
L*a*b*(L*)	71.979 ± 0.654	71.598 ± 0.869	71.427 ± 0.833	71.171 ± 1.240	72.113 ± 0.482	71.889 ± 0.633	71.886 ± 0.830	72.141 ± 0.598
L*a*b*(a*)	-0.841 ± 3.697	1.828 ± 3.826	4.475 ± 3.964	6.496 ± 6.259	-2.207 ± 2.368	-2.224 ± 1.858	-2.259 ± 1.923	-1.979 ± 3.126
L*a*b*(b*)	20.304 ± 5.065	20.015 ± 4.246	21.079 ± 5.425	23.103 ± 5.861	18.872 ± 4.639	19.708 ± 4.319	20.928 ± 4.902	20.658 ± 5.578
YCbCr(Y)	166.730 ± 1.231	166.275 ± 1.727	166.154 ± 1.734	165.560 ± 2.421	166.939 ± 1.016	166.373 ± 1.589	166.289 ± 1.921	166.921 ± 1.313
YCbCr(Cb)	110.228 ± 4.467	110.347 ± 3.762	109.288 ± 4.768	107.501 ± 5.147	111.558 ± 4.061	110.844 ± 3.791	109.768 ± 4.333	109.973 ± 4.920
YCbCr(Cr)	136.340 ± 4.116	138.785 ± 4.094	141.711 ± 4.428	144.367 ± 6.824	134.432 ± 2.858	134.748 ± 2.633	135.213 ± 2.830	135.377 ± 3.776

Abbreviations–RT_before_: before starting radiotherapy, RT_*n* days_: approximately *n* days after the first fraction of radiotherapy, RT_after_: approximately 10 days after radiotherapy completion.

To investigate the longitudinal changes in color space parameters within each group and between groups over time, the *p*-values were calculated via RM-ANOVA, as listed in S1 Table. For pairwise comparisons of the color space parameter values between irradiated and unirradiated breasts, the *p*-values of the Wilcoxon signed rank test were calculated for each longitudinal measurement point, as presented in Table 4. All color space parameters exhibited statistical significances of longitudinal changes in the values within and between groups over time, except for the b* value of the L*a*b* model and the Y and Cb values of the YCbCr model. Among these parameters, R of the RGB model, all parameter of the HSV model, a* of the L*a*b* model, and Cr of the YCbCr model showed statistically significant differences in the values between irradiated and unirradiated breasts for all longitudinal measurement points (all *p*-values <0.0167).

**Table 4:** *p*-values of Wilcoxon signed rank test for pairwise comparisons of color space parameter values between irradiated and unirradiated breasts for each longitudinal measurement point.

	RT_before_	RT_7 days_	RT_14 days_	RT_after_
RGB(R)^†^	-	< 0.001	< 0.001	< 0.001
RGB(G)^†^	-	0.004	< 0.001	< 0.001
RGB(B)^†^	0.007	-	-	< 0.001
HSV(H)^†^	-	< 0.001	< 0.001	< 0.001
HSV(S)^†^	-	0.001	< 0.001	< 0.001
HSV(V)^†^	-	< 0.001	< 0.001	< 0.001
L*a*b*(L*)^†^	-	-	-	0.002
L*a*b*(a*)^†^	-	< 0.001	< 0.001	< 0.001
L*a*b*(b*)	0.007	-	-	0.002
YCbCr(Y)	-	-	-	-
YCbCr(Cb)	0.006	-	-	0.001
YCbCr(Cr)^†^	-	< 0.001	< 0.001	< 0.001

Abbreviations–RT_before_: before starting radiotherapy, RT_*n* days_: approximately *n* days after the first fraction of radiotherapy, RT_after_: approximately 10 days after radiotherapy completion. Bonferroni adjustment. which considered the adjusted significance level of 0.0167, was applied for multiple comparisons.

^†^indicates statistical significances (*p* < 0.05) of longitudinal changes in the values of color space parameters within each group across time and differences in those for the interaction of time and group via a two-way repeated measures analysis of variance (RM-ANOVA).

### Spearman’s rank correlations

There were no statistically significant correlations between the values of the color space parameters and the RTOG scorings for the irradiated breasts.

In order to analyze the relationships between the values of the color space parameters and the skin dose values for the irradiated breasts, the correlation coefficients (*r*) with the corresponding *p*-values for Spearman’s rank correlation test were calculated; these are listed in Table 5. Only *r* values with *p*-values less than 0.05 are shown.

**Table 5:** Correlation coefficients (*r*) with corresponding *p*-values for Spearman’s rank correlation test between skin dose values and the values of the color space parameters for irradiated breasts.

	RT_7 days_		RT_14 days_		RT_after_	
	*r*	*p*	*r*	*p*	*r*	*p*
RGB(R)	0.583	0.008	0.567	0.010	0.499	0.027
RGB(G)	-	-	-	-	-	-
RGB(B)	-	-	-	-	-	-
HSV(H)	-	-	-	-	-	-
HSV(S)	0.450	0.048	0.450	0.048	0.484	0.032
HSV(V)	0.606	0.005	0.562	0.011	0.499	0.027
L*a*b*(L*)	-	-	-	-	-	-
L*a*b*(a*)	-	-	0.510	0.023	-	-
L*a*b*(b*)	-	-	-	-	-	-
YCbCr(Y)	-	-	-	-	-	-
YCbCr(Cb)	-	-	-	-	-	-
YCbCr(Cr)	-	-	0.609	0.005	0.552	0.013

*Abbreviations*: RT_*n* days_: approximately *n* days after the first fraction of radiotherapy, RT_after_: approximately 10 days after radiotherapy completion.

The Cr of the YCbCr model for RT_14 days_ was strongly correlated with the skin dose values (*r* = 0.690 with *p* < 0.005). The R of the RGB model and the S and V of the HSV model showed statistically significant correlations with the skin dose values for all three longitudinal measurement points (RT_7 days_, RT_14 days_, and RT_after_), with *r* > 0.450. Among these parameters, in general, the R of the RGB model, and the V of the HSV model exhibited stronger correlations with the skin dose values than the S of the HSV model did.

To determine the potential for predicting the severity of radiodermatitis early in the treatment course using color space parameters, the correlations among the values of these parameters for irradiated breasts at RT_after_, RT_7 days_, and RT_14 days_ were calculated. The *r* values and corresponding *p*-values obtained via the Spearman’s rank correlation test are presented in Table 6. All the color space parameters for the irradiated breasts at RT_7 days_ and RT_14 days_ showed moderate correlations with those at RT_after_, with *r* > 0.540 and *r* > 0.555, respectively, except for the L* of the L*a*b* model and the Y of the YCbCr model. In terms of correlations between RT_7 days_ and RT_after_ and between RT_14 days_ and RT_after_, the highest *r* values were observed for the b* of the L*a*b* model (*r* = 0.734 with *p* <0.001, and *r* = 0.895 with *p* < 0.001, respectively). Comprehensively, the strongest correlations among the values of the color space parameters at RT_after_ were observed for the R of RGB model and the S and V of the HSV model for both RT_7 days_ and RT_14 days_. However, the *r* values for RT_14 days_ vs. RT_after_ were higher than those for RT_7 days_ vs. RT_after_.

**Table 6:** Correlation coefficients (*r*) with corresponding *p*-values for Spearman’s rank correlation test between the values of color space parameters at approximately 10 days after finishing radiotherapy (RT_after_) and those at approximately 7 days after the first fraction of radiotherapy (RT_7 days_) and at RT_14 days_.

	RT_7 days_ vs. RT_after_		RT_14 days_ vs. RT_after_	
	*r*	*p*	*r*	*p*
RGB(R)	0.681	0.001	0.720	< 0.001
RGB(G)	0.540	0.015	0.555	0.012
RGB(B)	0.687	0.001	0.806	< 0.001
HSV(H)	0.586	0.008	0.710	< 0.001
HSV(S)	0.707	0.001	0.889	< 0.001
HSV(V)	0.690	0.001	0.713	< 0.001
L*a*b*(L*)	-	-	-	-
L*a*b*(a*)	0.588	0.007	0.684	0.001
L*a*b*(b*)	0.734	< 0.001	0.895	< 0.001
YCbCr(Y)	-	-	-	-
YCbCr(Cb)	0.716	0.001	0.892	< 0.001
YCbCr(Cr)	0.654	0.002	0.797	< 0.001

*Abbreviations*: RT_*n* days_: approximately *n* days after the first fraction of radiotherapy, RT_after_: approximately 10 days after radiotherapy completion.

## Discussion

In this study, we evaluated the performance of various color space models to assess the severity of the acute radiation dermatitis quantitatively. Furthermore, we adopted representative color space parameters to enable prediction of the early radiation-induced response of skin to overall treatment for radiodermatitis. To measure the skin color for both irradiated and unirradiated breasts, a mobile skin analysis device was used.

**Fig 1:**
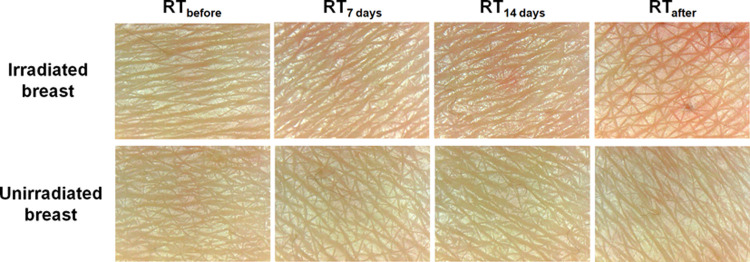
Skin images of both irradiated and unirradiated breasts acquired using a mobile skin analysis device during the course of the experiment. The skin images were acquired before radiotherapy (RT_before_), approximately 7 and 14 days after the first fraction of radiotherapy (RT_7 days_ and RT_14 days_), and approximately 10 days after completion of the radiotherapy (RT_after_).

**Fig 2:**
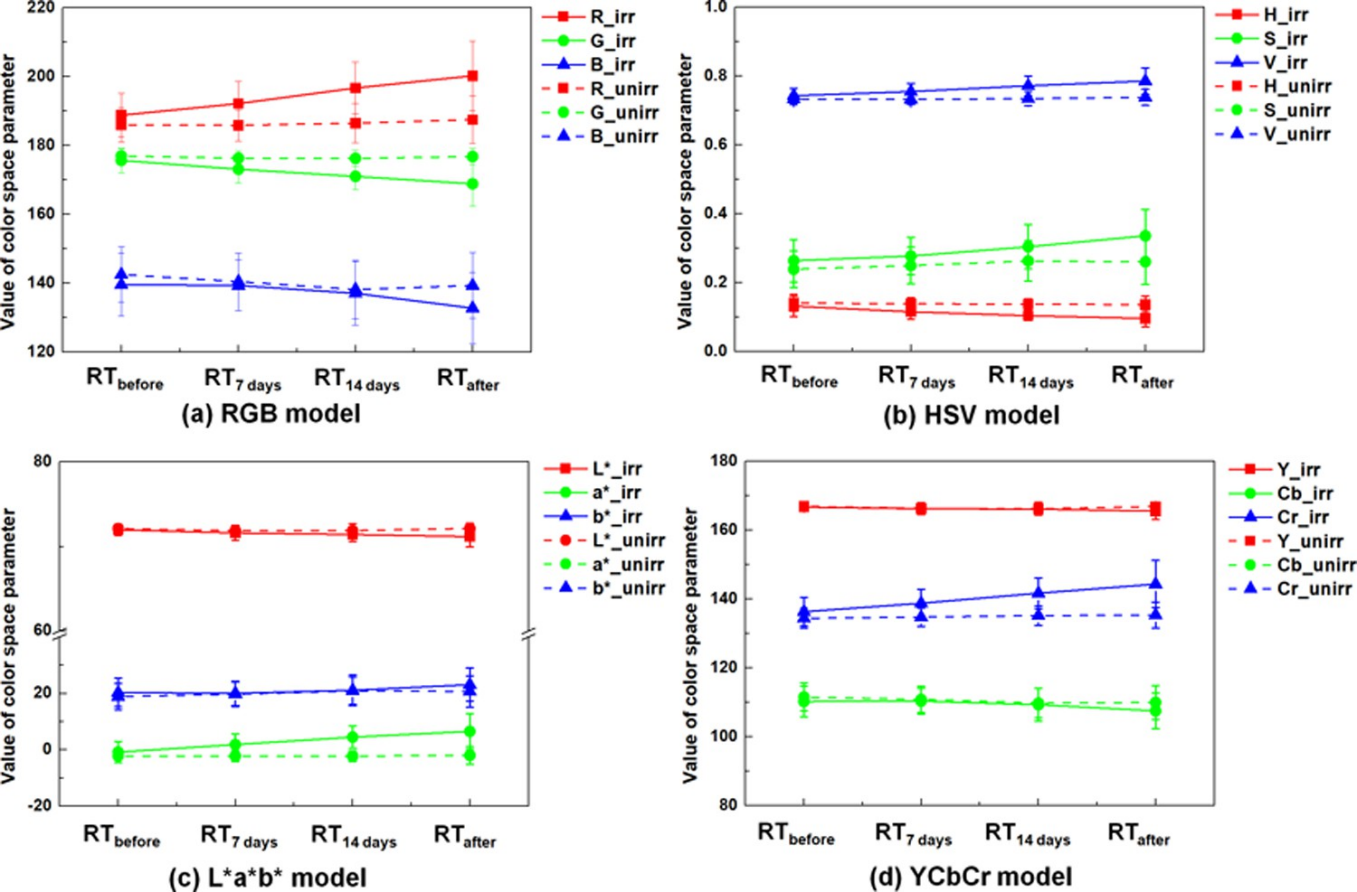
Mean and standard deviation of parameters for (a) RGB, (b) HSV, (c) L*a*b*, and (d) YCbCr color space models over time. “irr” and “unirr” denote the parameters for irradiated and unirradiated breasts, respectively.

Fig 1 shows skin images of both irradiated and unirradiated breasts obtained using the skin analysis device over the course of radiotherapy. As the skin was constantly damaged by radiation, the irradiated breasts became redder and darker than the unirradiated breasts. When comparing the skin images of the irradiated and unirradiated breast at RT_after_, we can easily observe the differences in skin color and then assess the severity of radiation dermatitis in the irradiated breasts subjectively. However, at RT_7 days_, the skin images for the irradiated and unirradiated breast were indistinguishable; thus, it was difficult to analyze the radiation-induced skin damage on the irradiated breasts through visual assessment. In this study, we analyzed all skin images for both sides of the breast by calculating various color space parameters. Fig 2 depicts the various temporal changes in the calculated parameters with four different color space models—RGB, HSV, L*a*b*, and YCrCb models. As radiotherapy progressed, significant increasing or decreasing tendencies in the color space parameters for the irradiated breasts were observed; these parameters peaked at RT_after_. Our results were largely consistent with those of other studies. Glover and Harmer reported that the severity of acute radiation dermatitis reaches its peak approximately 10–14 days after completion of radiotherapy of the breasts; thereafter, the severity gradually decreases as the epidermal basal layer recovers [22]. Other studies have experimentally demonstrated that radiotherapy gradually changes the skin color of irradiated breasts, as evidenced by increasing or decreasing representative parameters [14, 15, 21, 23]. The tendencies of the parameters for unirradiated breasts have been observed similar to those for irradiated breasts, but with minimal or no changes. These findings correspond well to those of earlier experimental studies [14, 15, 21, 23]. As shown in Figs 1 and 2, the skin images of the irradiated and unirradiated breasts at RT_7 days_, which are indistinguishable through visual inspection, exhibited significant differences in the values of some color space parameters. Thus, color space parameters can be considered appropriate for predicting or evaluating the severity of acute radiation dermatitis.

Several studies have reported similar results for the L*a* b* color space model used in our study. As the skin was damaged by radiation during radiotherapy, the L* values decreased (darker), whereas the a* values increased (redder), showing statistically significant correlations with physician-assessed scorings and doses [1, 13, 14, 23]. Böhner et al. conducted a prospective study on a large population of 142 Caucasian breast cancer patients and showed considerable correlations between the severity of radiodermatitis and objective L*a*b* color space model [12]. These studies have already validated the L*a*b* color space model in terms of reliability and usefulness in objective assessment of acute radiation dermatitis [1, 13, 14, 23]. However, no related studies that have employed the other color space models used in our study were found.

Our results showed statistically significant longitudinal changes with time in the color space parameters within each group as well as between the two groups. Moreover, noticeable correlations were observed between the color space parameters at different measurement points and between the parameters and skin dose values. Based on a comprehensive analysis, the R of the RGB model and the S and V of the HSV model achieved more noticeable performances than the other parameters did. The R values of the RGB model increased with time, showing the highest correlations for all statistical analysis, because these values plainly represent the red color in skin. The S and V values of the HSV model, which increased during radiotherapy, can represent pure red color (without any color components) and brighter colors, respectively. When considering the skin damage induced by the radiation, the increments in the S values were acceptable, whereas those in the V values could not be explained. As shown in the equation of V in the HSV model (S1 Appendix), the V values can be determined by obtaining the largest values among the R, G, and B of the RGB model. Therefore, when a particular color (e.g., red) becomes more prominent than others, the value of V increases. It was demonstrated that the degree of redness of skin due to radiation damage is greater than that of darkness of skin for acute radiation dermatitis.

The RGB, HSV, and YCbCr color space models, as well as the L*a*b* color space model, have been validated and used for skin color detection and segmentation. Shaik et al. presented a comparative study of human skin color recognition using the HSV and YCbCr color space models. They determined the threshold values for the individual components of corresponding color space parameters and demonstrated the suitable performance of the detection and segmentation skin pixels based on the determined threshold values [18, 19]. Rahmans et al. combined two color space models (HSV, and YCbCr) of skin color into a vector that contained the color elements of H, S, Cb, and Cr. They reported the combined model achieved significantly higher accuracy in terms of skin detection than the single color space models did [24]. Thus, various color space models can be useful for expressing skin color and texture, even in the case of radiodermatitis.

When considering that the pain of patients with acute radiation dermatitis not only significantly impacts the quality of life but can also delay standard treatment, prevention and management of radiodermatitis is an important aspect of radiotherapy. For preventing and treating acute radiation dermatitis, application of moisture cream, hyaluronic acid gel, and steroid ointment to skin damaged by radiation is recommended to provide moisture and protect the skin from developing secondary infections [25, 26]. Choi et al. evaluated the efficacy and safety of the use of the multi-lamellar emulsion for acute radiation dermatitis. This topical agent could be helpful to alleviate skin dryness and relieve radiodermatitis [27]. However, these products have their own characteristics such as color and gloss which can affect acquirement of skin images by a skin analysis device. Therefore, it is impossible to measure the true color and texture of the skin accurately. For this reason, we recommended no topical products for at least 8 h prior to measurement.

One limitation of the present study is the small number of samples restricted to one race of people (20 Asian patients for breast cancer) for statistical analysis. After the normality of the data was verified through the Shapiro-Wilk test, we conducted appropriate statistical tests for the non-parametric data. Depending on the race and ethnicity, the development of radiation-induced skin toxicity varied. Wright et al. reported that severe radiodermatitis was more commonly found in black patient than in white patients [28]. Although our study was only applied to Asian patients and can affect the results if applied to other races, similar or better performance of the color space model for evaluating radiodermatitis can be expected. However, further analysis with a large number of samples from various races must be performed to provide comprehensive applications and also ensure a high statistical certainty. Another limitation is that patients over grade +2 were not included. We were unable to assess the various skin reactions to radiodermatitis and the corresponding correlations. Nevertheless, strong correlations between the color space parameters and skin dose values were observed. It was demonstrated that the color space parameters have potential in evaluating and predicting slight differences in the severity of acute radiation dermatitis among all the enrolled patients within grade 1. Furthermore, we could measure the absolute doses for patients by only analyzing obtained skin images. If radiodermatitis is subdivided and then evaluated using the quantitative color space parameters proposed herein, proper management of skin reactions can be performed, and the effectiveness of radiotherapy can be improved. In future work, we intend to use large sample sizes and include different levels of severity of radiodermatitis, various races, and various treatment sites where acute radiation dermatitis can occur. Thus, we can expect to determine the tolerance level or action level of representative color space parameters for timely prevention and early diagnoses and treatment of radiodermatitis. In addition to statistical analysis of color space parameters, the use of machine learning techniques combined with such parameters will improve the evaluation and prediction of acute radiation dermatitis.

## Conclusions

In this study, we suggested a quantitative approach for evaluation of radiodermatitis in whole-breast radiotherapy, using various color space models. In general, the R of the RGB model and the S and V of the HSV model performed better than the other parameters did in evaluating acute radiation dermatitis. These color space parameters could be useful for evaluating the severity of radiodermatitis as they increase or decrease in a dose-dependent manner and also detect such manifestations which cannot be detected manually. This can be beneficial for patients undergoing radiotherapy since timely prevention and treatment of radiodermatitis will be ensured.

## Supporting information

S1 Appendix Equations used for calculating the color space parameters. (DOCX)

S1 Table *P*-values of a two-way repeated measures analysis of variance (ANOVA) in the three imaging modes. (DOCX)

S1 Data Raw data for color space parameters. (XLSX)

## References

[1] Cancer fact sheets: Breast cancer. International Agency for Research on Cancer (IARC). 2012. Available from: https://gco.iarc.fr/today/data/pdf/fact-sheets/cancers/cancer-fact-sheets-15.pdf.

[2] Sakyanun P, Saksornchai K, Nantavithya C, Chakkabat C, Shotelersuk K. The effect of deep inspiration breath-hold technique on left anterior descending coronary artery and heart dose in left breast irradiation. Radiat Oncol J 2020;38(3):181–188. doi: 10.3857/roj.2020.00094 33012146 PMC7533398

[3] Chen SN, Ramachandran P, Deb P. Dosimetric comparative study of 3DCRT, IMRT, VMAT, Ecomp, and Hybrid techniques for breast radiation therapy. Radiat Oncol J 2020;38(4):270–281. doi: 10.3857/roj.2020.00619 33389982 PMC7785843

[4] Delishaj D, D’amico R, Corvi D, Nobili GD, Alghisi A, Colangelo F, et al. Management of grade 3 acute dermatitis with moist desquamation after adjuvant chest wall radiotherapy: a case report. Radiat Oncol J 2020;38(4):287–290. doi: 10.3857/roj.2020.00983 33389984 PMC7785838

[5] International Agency for Research on Cancer. Breast cancer [Internet]. Lyon, France: International Agency for Research on Cancer; c 2020 [cited 2020 Nov 15]. Available from: https://gco.iarc.fr/today/fact-sheets-cancers. doi: 10.1007/s12105-019-01074-6

[6] Darby S, McGale P, Correa C, Taylor C, Arriagada R, Clarke M, et al. Effect of radiotherapy after breast-conserving surgery on 10-year recurrence and 15-year breast cancer death: meta-analysis of individual patient data for 10,801 women in 17 randomised trials. Lancet. 2011;378(9804):1707–1716. doi: 10.1016/S0140-6736(11)61629-2 22019144 PMC3254252

[7] Kim H, Park W, Kim SS, Ahn SJ, Kim YB, Kim TH, et al. Prognosis of patients with axillary lymph node metastases from occult breast cancer: analysis of multicenter data. Radiat Oncol J 2021;39(2):107–112. doi: 10.3857/roj.2021.00241 34619827 PMC8497863

[8] Huang CJ, Hou MF, Luo KH, Wei SY, Huang MY, Su SJ, et al. RTOG, CTCAE and WHO criteria for acute radiation dermatitis correlate with cutaneous blood flow measurements. Breast. 2015;24(3):230–236. doi: 10.1016/j.breast.2015.01.008 25777626

[9] Salvo N, Barnes E, van Draanen J, Stacey E, Mitera G, Breen D, et al. Prophylaxis and management of acute radiation-induced skin reactions: a systematic review of the literature. Curr Oncol. 2010;17(4):94–112. doi: 10.3747/co.v17i4.493 20697521 PMC2913836

[10] Bese NS, Sut PA, Sut N, Ober A. The impact of treatment interruptions on locoregional control during postoperative breast irradiation. J BUON. 2007;12(3):353–359. 17918289

[11] Schmeel LC, Koch D, Schmeel FC, Rohner F, Schoroth F, Bucheler BM, et al. Acute radiation-induced skin toxicity in hypofractionated vs. conventional whole-breast irradiation: An objective, randomized multicenter assessment using spectrophotometry. Radiother Oncol. 2020;146:172–179. doi: 10.1016/j.radonc.2020.02.018 32171945

[12] Bohner AMC, Koch D, Schmeel FC, Rohner F, Schoroth F, Sarria GR, et al. Objective Evaluation of Risk Factors for Radiation Dermatitis in Whole-Breast Irradiation Using the Spectrophotometric L*a*b Color-Space. Cancers (Basel). 2020;12(9):2444. doi: 10.3390/cancers12092444 32872216 PMC7563751

[13] Momm F, Bartelt S, Haigis K, Grosse-Sender A, Witucki G. Spectrophotometric skin measurements correlate with EORTC/RTOG-common toxicity criteria. Strahlenther Onkol. 2005;181(6):392–395. doi: 10.1007/s00066-005-1345-3 15925982

[14] Yamazaki H, Yoshida K, Kotsuma T, Kuriyama K, Masuda N, Nishimura T, et al. Longitudinal practical measurement of skin color and moisture during and after breast-conserving therapy: influence of neoadjuvant systemic therapy. Jpn J Radiol. 2009;27(8):309–315. doi: 10.1007/s11604-009-0345-0 19856226

[15] Yoshida K, Yamazaki H, Takenaka T, Tanaka E, Kotsuma T, Fujita Y, et al. Objective assessment of dermatitis following post-operative radiotherapy in patients with breast cancer treated with breast-conserving treatment. Strahlenther Onkol. 2010;186(11):621–629. doi: 10.1007/s00066-010-2134-1 21072624

[16] Gonzalez Sanchis A, Brualla Gonzalez L, Sanchez Carazo JL, Gordo Partearroyo JC, Esteve Martinez A, Vicedo Gonzalez A, et al. Evaluation of acute skin toxicity in breast radiotherapy with a new quantitative approach. Radiother Oncol. 2017;122(1):54–59 doi: 10.1016/j.radonc.2016.09.019 27825796

[17] Takiwaki H. Measurement of skin color: practical application and theoretical considerations. J Med Invest. 1998;44(3–4):121–126. 9597799

[18] Shaik KB, Ganesan P, Kalist V, Sathish BS, Jenitha JMM. Comparative study of skin color detection and segmentation in HSV and YCbCr color space. Procedia Comput Sci. 2015;57:41–48.

[19] Kaur A, Kranthi BV. Comparison between YCbCr color space and CIELab color space for skin color segmentation. Int J Appl Inf Syst. 2012;3(4):30–33.

[20] Kolkur S, Kalbande D, Shimpi P, Bapat J, Jatakia J. Human skin detection using RGB, HSV and YCbCr color models. International Conference on Communication and Signal Processing 2016 (ICCASP 2016). 2016.

[21] Kakumanu P, Makrogiannis S, Bourbakis N. A survey of skin-color modeling and detection methods. Pattern Recognit. 2007;40(3):1106–1122.

[22] Glover D, Harmer V. Radiotherapy-induced skin reactions: assessment and management. Br J Nurs. 2014;23(4):S28, S30–35. doi: 10.12968/bjon.2014.23.Sup2.S28 24619051

[23] Yamazaki H, Takenaka T, Aibe N, Suzuki G, Yoshida K, Nakamura S, et al. Comparison of radiation dermatitis between hypofractionated and conventionally fractionated postoperative radiotherapy: objective, longitudinal assessment of skin color. Sci Rep. 2018;8(1):12306. doi: 10.1038/s41598-018-30710-4 30120333 PMC6098151

[24] Rahman AM, Purnama KE, Purnomo MH. Simple method of human skin detection using HSV and YCbCr color spaces. 2014 International Conference on Intelligent Autonomous Agents, Networks and Systems. 2014.

[25] Kole AJ, Kole L, Moran MS. Acute radiation dermatitis in breast cancer patients: challenges and solutions. Breast Cancer: Targets Ther. 2017:9 313–323. doi: 10.2147/BCTT.S109763 28503074 PMC5426474

[26] Kwon MH, Yoon J, Kim EH, Lee Y, Yoon SW. A Literature Review of Management on Radiodermatitis. J Korean Tradit Oncol. 2020;25(1):11–24.

[27] Choi YW, Jung MJ, Son JH, Cho YS, Chung BY, Kim HO et al. Multi-lamellar emulsion(MLE)-based external application agents for patients with radiation dermatitis; a randomized case-control study. Korean J Dermatol. 2017;69(2):441.

[28] Wright JL, Takita C, Reis ID, Zhao W, Lee E, Hu JJ. Racial Variations in Radiation-Induced Skin Toxicity Severity: Data From a Prospective Cohort Receiving Postmastectomy Radiation. Int J Radiat Oncol Biol Phys. 2014;90(2):335–343. doi: 10.1016/j.ijrobp.2014.06.042 25304794

