# Supplementary material for: Quantitative evaluation of radiodermatitis following whole-breast radiotherapy with various color space models: A feasibility study
Source: PLoS One. 2022 Mar 9;17(3):e0264925. doi: 10.1371/journal.pone.0264925 (PMC8906630; doi:10.1371/journal.pone.0264925)
Supplement: S1 Appendix — (DOCX) [file pone.0264925.s001.docx]

**S1 Appendix**

**1. RGB to HSV conversion algorithm**

The R, G, and B values are divided by 255 to calculate the R, G, and B ratios (R’, G’, and B’) ranged from 0 to 1.

$$R^{'}= \frac{R}{255}$$

$$G^{'}= \frac{G}{255}$$

$$B^{'}= \frac{B}{255}$$

$$C_{max}=max(R^{'},G^{'},B^{'})$$

$$C_{min}=\min(R^{'},G^{'},B^{'})$$

- Hue (H) calculation:

$$\mathrm{Let}r= \frac{C_{max}-R'}{C_{max}-C_{min}};g= \frac{C_{max}-G'}{C_{max}-C_{min}}; b= \frac{C_{max}-B'}{C_{max}-C_{min}};$$

$$H^{'}= \left\{ \begin{aligned} 5+b, if R^{'}= C_{max} and G^{'}= C_{min} \\ 1-g, if R^{'}= C_{max} and B^{'}= C_{min} \\ 1+r, if G= C_{max} and B^{'}= C_{min} \\ 3-b, if G^{'}= C_{max} and R^{'}= C_{min} \\ 3+g, if B^{'}= C_{max} and R^{'}= C_{min} \\ 5-r, if B^{'}= C_{max} and G^{'}= C_{min} \end{aligned} \right.$$

$$H=\frac{H^{'}}{6}$$

- Saturation (S) calculation:

$$S= \left\{ \begin{aligned} 0 , C_{max}=0 \\ \frac{C_{max}-C_{min}}{C_{max}}, C_{max}\neq0 \end{aligned} \right.$$

- Value (V) calculation:

$$V = C_{max}$$

**2. RGB to L*a*b* conversion algorithm**

Step 1: Conversion from RGB to CIE XYZ

$$X=0.57667\cdot{R^{'}}^{2.1992}+0.18555\cdot{G^{'}}^{2.1992}+0.18819\cdot{B^{'}}^{2.1992}$$

$$Y=0.29738\cdot{R^{'}}^{2.1992}+0.62735\cdot{G^{'}}^{2.1992}+0.07527\cdot{B^{'}}^{2.1992}$$

$$Z=0.02703\cdot{R^{'}}^{2.1992}+0.07069\cdot{G^{'}}^{2.1992}+0.99110\cdot{B^{'}}^{2.1992}$$

Step 2: Conversion from CIE XYZ to CIE L*a*b*

Reference X, Y, and Z values of standard illuminants (Ref_X, Ref_Y, and Ref_Z) were obtained by using Matlab ‘whitepoint’ function. For this condition, the Ref_X, Ref_Y, and Ref_Z were 0.9642, 1.0000, and 0.8249, respectively.

$$X^{'}= \left\{ \begin{aligned} {(\frac{X}{Ref\_X})}^{1/3}, if\frac{X}{Ref\_X}>0.008856 \\ 7.787\cdot\frac{X}{Ref\_X}+\frac{16}{116}, if\frac{X}{Ref\_X}\leq0.008856 \end{aligned} \right.$$

$$Y^{'}= \left\{ \begin{aligned} \left( \frac{Y}{Ref\_Y} \right)^{\frac{1}{3}}, if\frac{Y}{Ref\_Y}>0.008856 \\ 7.787\cdot\frac{Y}{Ref\_Y}+\frac{16}{116}, if\frac{Y}{Ref\_Y}\leq0.008856 \end{aligned} \right.$$

$$Z^{'}= \left\{ \begin{aligned} {(\frac{Z}{Ref\_Z})}^{1/3}, if\frac{Z}{Ref\_Z}>0.008856 \\ 7.787\cdot\frac{Z}{Ref\_Z}+\frac{16}{116}, if\frac{Z}{Ref\_Z}\leq0.008856 \end{aligned} \right.$$

CIE L*, a*, and b* can be calculated as follows:

$$L^{*} =\left( 116\cdot Y^{'} \right)-16$$

$$a^{*}=500\cdot(X^{'}-Y^{'})$$

$b^{*}=200\cdot(Y^{'}-Z^{'})$

**3. RGB to YCbCr conversion algorithm**

$$Y=16+(65.481\cdot R^{'}+128.553\cdot G^{'}+24.966\cdot B^{'})$$

$$C_{b}=128+(-37.797\cdot R^{'}-74.203\cdot G^{'}+112.0\cdot B^{'})$$

$$C_{r}=128+(112.0\cdot R^{'}-93.786\cdot G^{'}-18.214\cdot B^{'})$$
