# Supplementary material for: Quantitative evaluation of radiodermatitis following whole-breast radiotherapy with various color space models: A feasibility study
Source: PLoS One. 2022 Mar 9;17(3):e0264925. doi: 10.1371/journal.pone.0264925 (PMC8906630; doi:10.1371/journal.pone.0264925)
Supplement: S1 Table — (DOCX) [file pone.0264925.s002.docx]

S1 Table. *P*-values of a two-way repeated measures analysis of variance (ANOVA) in the three imaging modes

|  | Time | Time:group |
| --- | --- | --- |
| RGB(R) | < 0.001 | < 0.001 |
| RGB(G) | < 0.001 | < 0.001 |
| RGB(B) | 0.001 | < 0.022 |
| HSV(H) | < 0.001 | < 0.001 |
| HSV(S) | < 0.001 | 0.006 |
| HSV(V) | < 0.001 | < 0.001 |
| LAB(L) | 0.003 | 0.021 |
| LAB(A) | < 0.001 | 0.008 |
| LAB(B) | - | - |
| YCbCr(Y) | - | - |
| YCbCr(Cb) | 0.004 | - |
| YCbCr(Cr) | < 0.001 | < 0.001 |

*Abbreviations*: UV = ultraviolet; IMC = inverse measure of correlation.
